# More insights into acute aortic dissection, but with what clinical consequence?

**DOI:** 10.1093/icvts/ivac052

**Published:** 2022-03-07

**Authors:** Martin Misfeld

**Affiliations:** 1 University Department of Cardiac Surgery, Leipzig Heart Center, Leipzig, Germany; 2 Department of Cardiothoracic Surgery, Royal Prince Alfred Hospital, Sydney, NSW, Australia; 3 Institute of Academic Surgery, RPA, Sydney, NSW, Australia; 4 The Baird Institute of Applied Heart and Lung Surgical Research, Sydney, NSW, Australia; 5 Sydney Medical School, The University of Sydney, Sydney, NSW, Australia

**Keywords:** Aorta, Aortic dissection, Biochemistry, Biomechanics

The manuscript ‘Rapid evaporative ionisation mass spectrometry (Intelligent Knife) for point-of-care testing in acute aortic dissection surgery’ by Davies *et al.* [1] in this issue describes a small series of 10 patients, in which the smoke, generated by the use of a monopolar diathermy electrosurgical pencil was immediately analysed in addition to further biomechanical and biochemical analyses. Tissue samples were divided into dissection-flap, true lumen and false lumen samples, respectively. Same patterns were classified with 72.4% accuracy and 69.3% precision. The authors conclude that their findings could dictate future clinical distal disease progression and may alter intraoperative decision-making.

Although extensive histological and clinical studies have been performed in patients with acute aortic dissection (AAD), mechanisms of onset of AAD, risk factors of progression and best surgical treatment remain to be under debate. This is attributed to the fact, that AAD represent not a single pathology, but a clinical occurrence of a complication of different underlying pathologies (e.g. connective tissue disorders, bicuspid aortic valves, various aneurysm formations, positive family history, etc.). What is known is that the aorta changes with age [2] and that there are specific gender differences in AAD [3]. Therefore, the relatively small number of patients in this study, the ratio male:female of 7:3, as well as the age range between 27 and 75 years may represent different biochemical and biomechanical findings of the aortas itself.

Rapid evaporative ionization mass spectrometry is a fascinating and intriguing tool and together with the sophisticated further analyses the authors conducted, we get more insight into the pathology of AAD. However, ‘prediction’ is the key question. ‘Prediction’ of either preventing AAD or progression of further aortic disease. The authors state that the extent of the surgical procedure during treatment of AAD may be modified with regard to the rapid analysis. However, as the occurrence of the extend and potential complications of AAD is so heterogenous, the primary goal must be to treat the acute stage with patient survival. This leads to 2 basic operative strategies: (i) Treating as much as necessary on the one hand and getting the patient out of the life-threatening situation or (ii) Doing as much as possible (e.g. complete arch with frozen elephant trunk) preventing further disease progression. The authors also speculate that disease progression may be calculated. I doubt that from the results given this conclusion can be made. It remains speculative unless they correlate their initial finding with more patients with future imaging results, as they announce to do so.

The gold standard for disease progression is and will be a close surveillance with magnetic resonance or computer tomography imaging evaluated by dedicated aortic centres. It also seems to be of more interest, being able to predict and, therefore prevent AAD. It is well known that AAD occurs not only after the recommended diameters of aneurysm have been passed [4]. We also know that during dissection, the aorta increases in diameter itself [5]. Therefore, it seems to be of immense importance to identify risk groups and further predict their risk before an AAD occurs [6].

Understanding the disease is important. This will lead to potential disease prevention before a complication occurs. As we have not reached this ability of disease prevention, further disease progression is of additional interest. We are learning more and have gained more insight into this in the past years.

The study by Davies contributes to disease knowledge, but is not able to contribute to understand disease progression and least of all to disease prevention.
